# Tumor-to-tumor metastasis of breast carcinoma to clear cell renal cell carcinoma: a rare case with a review of the literature

**DOI:** 10.1007/s13730-026-01156-9

**Published:** 2026-07-28

**Authors:** Nobuyuki Nakajima, Hiroshi Kajiwara, Kenta Okutsu, Toshiki Sakemura, Haruka Ikoma, Tatsuya Otaki, Soichiro Yuzuriha, Takato Uchida, Tatsuya Umemoto, Yoshiaki Kawamura, Yohei Masugi, Sunao Shoji

**Affiliations:** 1 Department of Urology, Tokai University School of Medicine, 143 Shimokasuya, Isehara, 259-1193 Kanagawa Japan; 2 Department of Pathology, Tokai University School of Medicine, 143 Shimokasuya, Isehara, 259-1193 Kanagawa Japan

**Keywords:** Breast carcinoma, Clear cell renal cell carcinoma, Tumor-to-tumor metastasis, Immunohistochemistry, Histopathology

## Abstract

Tumor-to-tumor metastasis (TTM) is a rare clinicopathological phenomenon in which a malignant tumor metastasizes to another distinct neoplasm. Although renal cell carcinoma (RCC) is a frequent recipient tumor, metastasis from breast carcinoma to RCC is extremely rare. A 64-year-old woman with long-standing hormone receptor–positive breast carcinoma presented with a 23-mm enhancing right renal mass. Robot-assisted partial nephrectomy revealed clear cell RCC with intratumoral nests of metastatic breast carcinoma. Immunohistochemistry revealed a reciprocal staining profile: RCC cells were positive for PAX8 and CA9, whereas metastatic breast carcinoma cells expressed CK7 and GATA3 with weak ER positivity. Clear cell RCC may serve as a recipient tumor for breast carcinoma metastasis, emphasizing that TTM should be considered in the differential diagnosis of renal masses in patients with a history of breast carcinoma, even when imaging findings are consistent with conventional RCC.

## Introduction

Tumor-to-tumor metastasis (TTM) is an uncommon metastatic pattern in which a malignant tumor metastasizes to another distinct neoplasm. Campbell et al. first summarized this phenomenon in 1968 [1], and the subsequent diagnostic criteria have emphasized the confirmation of two independent neoplasms and the exclusion of collision, direct extension, and tumor emboli [1, 2]. Among malignant recipient tumors, renal cell carcinoma (RCC) is disproportionately represented [2, 3], likely owing to its hypervascularity, lipid- and glycogen-rich cytoplasm, and permissive stromal microenvironment. Breast carcinoma is a recognized donor; however, metastasis specifically to the RCC is exceedingly rare. Since 2006, eleven cases have been reported worldwide [4–14], including one from Japan [9]. Herein, we report a rare case of a Japanese patient confirmed using contemporary histopathological and immunophenotypic criteria.

## Case presentation

**Fig. 1:**
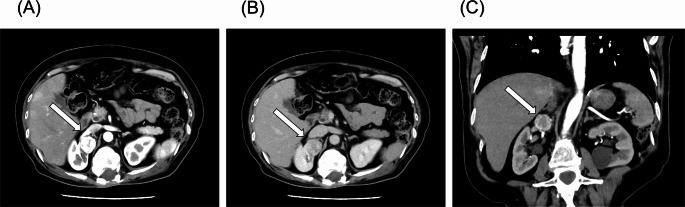
Contrast-enhanced computed tomography findings of the right renal mass. **A** Axial corticomedullary phase demonstrating a strongly enhancing 23-mm mass in the right kidney, **B** Axial equilibrium phase showing persistent enhancement without evidence of central necrosis, **C** Coronal reconstruction illustrating the anatomical location of the tumor within the right kidney

A 64-year-old Japanese woman with a history of invasive ductal carcinoma of the left breast diagnosed in 2007 was evaluated for a newly identified right renal mass. The primary breast carcinoma was estrogen receptor-positive (Allred 5 + 2), progesterone receptor-positive (Allred 4 + 2), and HER2 immunohistochemistry score 2+. Bone metastases developed in 2011, and the patient subsequently received multiple lines of endocrine therapies. At the time the renal lesion was detected in 2025, she was receiving systemic therapy with fulvestrant and capivasertib. At that time, metastatic disease was confined to bone, without known visceral metastases. Recent surveillance imaging had shown no evidence of systemic progression. The renal lesion was detected on routine contrast-enhanced CT performed as part of oncologic follow-up rather than prompted by new symptoms or suspected disease progression. Contrast-enhanced CT demonstrated a solitary 23-mm well-circumscribed mass in the right kidney. On corticomedullary phase imaging, the lesion showed avid early enhancement relative to the surrounding renal parenchyma. On equilibrium phase imaging, the lesion remained enhancing without evidence of central necrosis or hemorrhage (Fig. 1). The mass appeared predominantly homogeneous and did not demonstrate invasion of the collecting system or major renal vessels. No suspicious lymphadenopathy or additional metastatic lesions were identified at that time.

Given the solitary nature of the lesion and radiologic features suggestive of conventional clear cell RCC, the tumor was considered surgically resectable. Therefore, percutaneous renal mass biopsy was not pursued, and robot-assisted laparoscopic partial nephrectomy was performed without perioperative complications. Gross examination revealed a yellowish solid tumor without features indicative of metastasis or necrosis.

Histopathological examination revealed that most of the tumor consisted of clear cell RCC; however, distinct foci of atypical epithelial cells with duct-forming structures were also observed. Microscopically, the metastatic breast carcinoma formed discrete intratumoral tumor nests within the clear cell RCC, accompanied by stromal infiltration. Although the boundary between the two components was predominantly sharp, limited transitional areas were observed focally. The metastatic component was not confined to vascular spaces; rather, it formed a mass with stromal invasion, arguing against isolated intravascular tumor emboli. Desmoplastic reaction was not identified. On low-power examination, the metastatic breast carcinoma component accounted for approximately 30% of the tumor area. Focal lymphovascular invasion was identified in both components, slightly more prominent in the metastatic breast carcinoma. The clear cell RCC component was classified as pT1a and WHO/ISUP grade 1 with focal grade 2 nuclear features, without capsular invasion. The surgical margins were negative.

**Fig. 2:**
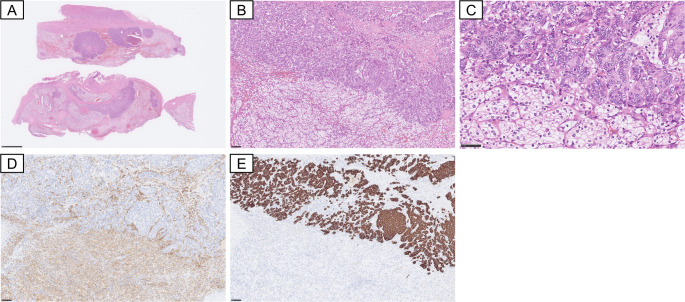
Histopathological and immunohistochemical findings of the renal tumor. **A **Whole-slide hematoxylin and eosin (H&E) image showing clear cell renal cell carcinoma containing discrete intratumoral nests of metastatic breast carcinoma. **B** Low-power H&E staining (×50) showing clear cell renal cell carcinoma (RCC) occupying the lower portion of the tumor, with discrete intratumoral nests of metastatic breastcarcinoma in the upper portion. **C** High-power H&E staining (×200) demonstrating the distinct cytological features of the two components: clear cytoplasm and delicate vasculature in the RCC and crowdedatypical ductal structures in the metastatic breast carcinoma. **D** CA9 immunostaining showing strong membranous positivity confined to the clear cell RCC component, with complete absence of staining in the metastatic breastcarcinoma nests. **E** GATA3 immunostaining highlighting the metastatic breast carcinoma component with diffuse nuclear positivity, while the adjacent RCC component remains entirely negative,demonstrating the reciprocal immunophenotype characteristic of tumor-to-tumor metastasis

Immunohistochemistry confirmed a reciprocal staining profile: RCC cells were positive for PAX8 and CA9 and negative for CK7, GATA3, and hormone receptors, whereas the metastatic breast carcinoma component showed CK7 and GATA3 positivity with weak ER expression and was negative for PAX8 and CA9. The ER status was concordant with that of the primary breast carcinoma (Allred 5 + 2), although with lower staining intensity (Fig. 2).

The postoperative course was uneventful, and renal function remained stable. The patient was followed at our institution for 1 month without surgical complications. Thereafter, oncologic follow-up was continued at the referring hospital. Following histopathological confirmation that the renal lesion represented metastatic breast carcinoma within clear cell RCC, the breast carcinoma was considered to meet criteria for systemic progression. At the time of surgery, no additional visceral metastases had been identified. Accordingly, a transition to trastuzumab deruxtecan–based therapy was planned.

## Discussion

TTM is an uncommon but well-established pathological condition. The first comprehensive diagnostic framework was proposed in the mid-20th century by Campbell et al. [1]. Subsequent clinicopathological reviews have refined this definition and catalogued reported cases across diverse primary and recipient tumor types [2]. According to these established criteria, a diagnosis of TTM requires the presence of two histologically distinct primary tumors, evidence of true metastatic growth within the recipient neoplasm, and exclusion of collision tumors, direct extension, or tumor embolization. In the present case, the metastatic breast carcinoma component was clearly demarcated within the clear cell RCC and demonstrated a reciprocal immunophenotypic profile (PAX8/CA9-positive RCC cells and CK7/GATA3-positive breast carcinoma cells), supporting a true TTM process rather than a simple juxtaposition of two independent neoplasms. In addition, focal transitional areas were observed microscopically, and the metastatic component demonstrated stromal infiltration rather than confinement to vascular spaces, arguing against collision tumor or intravascular tumor emboli. The metastatic breast carcinoma component accounted for approximately 30% of the tumor area, further supporting true intratumoral metastatic growth. A recent systematic evaluation identified more than 600 documented TTM events, highlighting that although rare, TTM is a reproducible biological and clinical phenomenon [3].

Breast carcinoma is one of the most frequently reported donor tumors in TTM, a finding that has been attributed, in part, to its global incidence and prolonged natural history, which together increase the temporal opportunity for rare metastatic events [3]. In our focused review, among breast carcinomas reported as donor tumors in TTM involving RCC since 2006, both invasive ductal and invasive lobular carcinomas have been described [4–14]. These observations indicate that breast carcinoma can act as a donor tumor in TTM across different histological subtypes, although the relative contribution of tumor biology as opposed to incidence and survival remains unclear.

Clear cell RCC is the most common malignant tumor reported in TTM recipients [2, 3], and several hypotheses have been proposed to explain this susceptibility. The kidney, particularly clear cell RCC, is characterized by abundant sinusoidal vasculature, lipid- and glycogen-rich cytoplasm, and a stromal microenvironment capable of supporting foreign tumor cell engraftment [2]. In addition, experimental studies have demonstrated that the tumor microenvironment, shaped by immunoregulatory mechanisms such as tumor-associated macrophages and vascular endothelial growth factor (VEGF)-driven angiogenesis, can promote immune suppression and facilitate metastatic cell survival [15].

Collectively, these biological characteristics are consistent with Paget’s classical “seed and soil” theory, which proposes that metastatic dissemination depends on the compatibility between disseminated tumor cells (“seeds”) and the microenvironment of the target organ (“soil”) [16]. This concept was subsequently refined by studies emphasizing organ-specific interactions mediated by growth factors, chemokines, signaling pathways, and gene expression programs, which contribute to metastatic organ preference [17].

In the present case, the coexistence of breast carcinoma cells within a vascular and metabolically permissive clear cell RCC microenvironment provides a clinicopathological illustration of this concept.

In line with this theoretical framework, multiple clinicopathological reports have documented breast carcinoma metastasizing into RCC. Since 2006, 11 well-documented cases have been reported in the modern literature [4–14]. Including the present case, a total of 12 cases are summarized in Table 1. A recent systematic review identified 28 breast-to-kidney TTM events [3]; however, many earlier reports predated routine immunohistochemistry and contemporary RCC classification, which may limit diagnostic certainty. For clinicopathological consistency, we focused on cases published since 2006 that were histologically confirmed with clear immunohistochemical distinction between donor and recipient components. The restriction to reports published after 2006 was applied to enhance clinicopathological consistency, as routine immunohistochemical characterization and contemporary RCC classification became more widely adopted during this period. These include classical invasive ductal carcinoma [4, 6–9, 11–13], invasive lobular carcinoma [10], and rare subtypes, such as neuroendocrine breast carcinoma [5]. Collectively, these cases reinforce that donor tumor histology alone does not determine metastatic potential; rather, the capacity to adapt to the metabolic and immunological landscape of RCC appears to be central to successful engraftment. Notably, metastasis has also been described within an RCC pulmonary metastasis [11], illustrating that TTM may occur not only within primary tumors but also in metastatic sites.

**Table 1:** Reported cases of tumor-to-tumor metastasis from breast carcinoma to renal cell carcinoma since 2006

No.	Author / Year	Donor breast carcinoma	RCC subtype	RCC size	Surgery	Laterality	Key IHC findings
1	Möller et al., 2006[4]	IDC	Clear cell	3 cm	Radical nephrectomy	Right	Breast: ER+, PgR + RCC: NR
2	Ulamec et al., 2008[5]	Neuroendocrine breast carcinoma	Clear cell	11 cm	Nephrectomy	NR	Breast: ER+, PgR+ Synaptophysin+; RCC: CD10+, vimentin+, AE1/AE3-
3	Huo et al., 2015[6]	IDC	Clear cell	3.0 cm	Partial nephrectomy	Left	Breast: ER+, Mammaglobin+, CK7+; RCC: CD10+, Vimentin+, ER-
4	Gazel et al., 2018[7]	IDC	Clear cell	2.7 × 2.8 × 3.3 cm	Radical nephrectomy	Left	Breast: ER+, GATA3+, GCDFP-15+; RCC: PAX8+
5	Lakovschek et al., 2019[8]	IDC	Clear cell	6.5 cm	Radical nephrectomy	Right	Breast: ER+, GATA3+; RCC: CD10+, GATA3-, ER-
6	Shibata et al., 2019[9]	IDC	Clear cell	5.3 cm	Radical nephrectomy	Left	Breast: ER+, GCDFP+ ; RCC: NR
7	Ashman et al., 2020[10]	ILC	Clear cell	NR	NA	Right	Breast: ER+, GATA3+, E-cadherin-; RCC: PAX8+, GATA3-
8	Lima et al., 2021[11]	IDC	Pulmonary RCC metastasis	NR	Metastasectomy		Breast: ER+, Cam5.2 +, CK7 +; RCC: CD10+, Vimentin+, ER-
9	Khan et al., 2023[12]	IDC	Clear cell	NR	Radical nephrectomy	Left	Breast: ER-, PR-, HER2-, AE1/AE3+ ; RCC: NR
10	Hau et al., 2024[13]	IDC	Clear cell	3.3 cm	Partial nephrectomy	NR	Breast: GATA3+; CK7+, ER+, PR+; RCC: PAX8+, CAIX+, CD10+
11	Murugesan et al., 2025[14]	NR	Clear cell	3.0 × 1.6 cm	NA	Right	Breast: GATA3+, ER+, mammaglobin+, CK7+; RCC: CA9+, CD10+, PAX8+
12	Present case, 2025	IDC	Clear cell	2.3 cm	Robot-assisted partial nephrectomy	Right	Breast: CK7+, GATA3+; RCC: PAX8+, CA9+

Abbreviations: TTM, tumor-to-tumor metastasis; RCC, renal cell carcinoma; IDC, invasive ductal carcinoma; ILC, invasive lobular carcinoma; ER, estrogen receptor; PR, progesterone receptor; CAIX/CA9, carbonic anhydrase IX; NR, not reported; NA, not applicable

Radiologically, distinguishing TTM from primary RCC is challenging. While clear cell RCC typically presents as a hypervascular, well-circumscribed mass, breast-derived metastatic foci may appear infiltrative or heterogeneous. In the present case, the lesion demonstrated imaging characteristics typical of conventional clear cell RCC, without overt radiologic features suggestive of metastatic disease. Several reports have described renal masses that were clinically diagnosed as RCC, with TTM identified only on histopathological examination, demonstrating dual tumor populations [4–14]. Thus, when RCC is identified in patients with a history of breast carcinoma, renal biopsy may aid in the differential diagnosis. However, in the context of TTM, a small metastatic component within a dominant RCC may be missed because of sampling limitations.

Pathologically, TTM diagnosis requires the identification of two distinct neoplastic populations within the same mass and donor–recipient differentiation through immunohistochemistry. The consistently reported immunoprofiles—breast carcinoma markers such as GATA3, ER/PgR, CK7, and mammaglobin, alongside RCC markers such as PAX8, CA9, and CD10—have been central to establishing the diagnosis in the literature [4–14]. The present case also demonstrated classical dual-staining patterns consistent with TTM. In addition, ER expression was weakly retained in the metastatic component and was concordant with the ER-positive status of the primary breast carcinoma, although with reduced staining intensity. This finding argues against complete receptor conversion in this case. In addition to its diagnostic relevance, TTM may provide a unique and naturally occurring model for studying metastatic biology. Unlike experimental systems, TTM represents a real-world environment in which circulating tumor cells colonize and survive in an established malignant microenvironment. Mechanistic insights from tumor ecology, microenvironmental immunology, and metastatic niche biology [15, 17, 18] may help contextualize TTM as a naturally occurring model of metastatic niche selection within an established malignant microenvironment.

Clinically, the recognition of TTM is important because misclassification may lead to inaccurate staging or inappropriate assumptions about intratumoral heterogeneity or sarcomatoid transformation. Although surgical management is generally dictated by the biological behavior of the recipient RCC, distinguishing TTM from primary or metastatic RCC may influence systemic therapy decisions and multidisciplinary planning.

## Conclusion

In conclusion, this case demonstrates that clear cell RCC can serve as a recipient tumor for breast carcinoma metastasis. TTM should be considered in the differential diagnosis of renal masses in patients with a history of breast carcinoma, even when the imaging findings are consistent with conventional RCC.

## Data Availability

All data generated or analyzed during this study are included in this article.
